# St. Thomas's Hospital and Medical School

**Published:** 1906-07-07

**Authors:** 


### HOSPITAL ADMINISTRATION.

### CONSTRUCTION AND ECONOMICS.

### ST. THOMAS'S HOSPITAL AND MEDICAL SCHOOL.

### PROFESSOR OSLER, M.D., F.R.S., ON ITS WORK.

After the distribution of prizes at St. Thomas's Hospital last week Professor Osler delivered the following address:—

It gives me great pleasure to be at such a gathering. My associations with this hospital are very close, and my earlier recollections as a student in Toronto are of men who had been trained at St. Thomas's. This was for many years the one great hospital for Canadians for post-graduate work, and I should like to express the thanks of Canada for the great kindness to Canadian students in association with this school. When I returned to England in 1878 Dr. Murchison was kind enough to allow me to frequent his clinical classes, so for three months I walked the wards of this hospital with him, and I have therefore many friends on the staff.

Finance and Pay Wards.

I have to congratulate you on the remarkable financial condition of the hospital. I have been looking at the balance-sheets of the other great London hospitals, and there is no other with one like yours. You are to be congratulated that you have so small a balance on the wrong side. You have one feature which interests me particularly, and that is that you admit a certain number of pay-patients, and I see you made £800 by them last year. They come from the ranks of comparatively poor people, and I want to plead for the poor rich, who support the hospitals and who do not get nearly as much out of the hospitals as they should. I am a great believer in pay-wards in general hospitals; for there is no place where people can get the same care and forethought during sickness and convalescence as in a general hospital. I should like to see in connection with every great general hospital a large pay-pavilion established, and in saying so I speak after considerable thought and experience in connection with the Johns Hopkins Hospital. The work of pay-wards is not only very interesting, but it is delightful to see how in a few years people have got into the habit of coming to be treated in the private wards of the hospitals. In the large towns of the United States it is the common practice for well-to-do people to go there for operations and for serious illnesses. I cannot help thinking how much good it would do the Lady Clara Vere de Vere if, when she had appendicitis, she were to come to this hospital and during her convalescence to wander up and down the wards and see the poor patients here.

Clinical and Surgical Research.

One other thing I may congratulate you on, your development of research on the clinical and surgical side. Only in that way can progress be made. This hospital was one of the first to establish a clinical laboratory, and in that connection I should like to read some extracts from a letter sent with a gift for the Lambert Fund for the encouragement of research. The writer says:—

"I want in the first place to express satisfaction at the foundation of the Fund and at the circumstances under which this foundation has occurred. It is, as I understand, an expression of gratitude on the part of the donor—who is not a member of the medical profession nor a scientist—for services which have been rendered to a patient in St. Thomas's by a member of the hospital staff in conjunction with the director of the hospital laboratories. Those associated in the government of St. Thomas's have, it is true, appreciated the close connection between the welfare of the patients and the scientific work carried on in the laboratory. You have in fact given practical proof of your appreciation by the establishment of these very laboratories. It is in consequence of this action of yours that we can now claim that for every patient within the walls of St. Thomas's there is available an investigation as complete as modern methods can make it and a treatment which is correspondingly effective.

"Unfortunately this view of the bearing of scientific research upon practical medicine is one which meets with but limited acceptance in the outside world, and only too often its upholders are visited with a contumely which is shared by any institution with which they may be connected. That this fund for the promotion of scientific research should have been inaugurated by a layman, and under such circumstances seems to me a most auspicious and significant fact which must bring peculiar gratification to you and your colleagues as a spontaneous testimony to the wisdom of your administration.

"And now to the second object of this letter; for, as you have probably guessed, it is not written merely to congratulate St. Thomas's on what has already been achieved. This second object is to carry the ideal of hospital efficiency a stage further, and to do so by calling to our aid that form of philanthropy (best seen in the provinces and the United States) which makes science its servant and is guided by its head no less than by its heart.

"A hospital should be regarded as an institution not only for relieving those who are already sick, but for combating disease. Unless this is realised no great success can be expected or will be attained any more than would be the case in a war waged by an army consisting of an ambulance service without a fighting line. In the war against disease the ambulance service, represented by our wards and out-patient department, is as much a necessity as ever; the value of this service is obvious and it stands in no danger of neglect. But it is far otherwise with the fighting line in the laboratory. Very few regard that as philanthropy, and yet what are the facts? Through Behring's work in the laboratory diphtheria has been robbed of most of its terrors. Pasteur, who was not even a medical man, has freed the world to a very great extent from the dread of hydrophobia. Koch has revealed the cause of consumption, and has led an advanced band far on the road towards the cure of that fell disease. But there is no need to multiply instances to prove that life-saving on the great scale goes on in the laboratory rather than in the ward. And yet what has been achieved is nothing to what may be anticipated in the future. Hope is in the air and has already found its justification in success attained. In all time there has never been an opportunity like the present for a far-seeing philanthropy devoted to the support of scientific investigation. In the past science has wandered in a maze, knowing only the results of disease. Then, through the development of bacteriology, it came to close grips with its causes. Now it is wrestling with the problem of cure. Thus it comes about that the most noteworthy feature of research at the present day is its intense practicality."

A Word to the Students.

And now students of medicine a word to you. The prizes you have just received have been fought for with no little effort; go on with more efforts. But since all of you are young remember what Plato once said of a man of his acquaintance: "Ah! he didn't attain success; he went too fast when he was young." So I urge you not to go too fast. The best success is attained by steady, slow effort. To my mind there is only one text for medical students: "Take no thought for to-morrow." That is the only motto worth putting up in your rooms. Have no fear about your examinations. You have only the day's work to do, and do that well. Carlyle once said, "Our business is not to look at what lies dimly in the future, but at what lies close at hand." Look to your precious time. There is not one of us who has not to look back with regret on wasted hours. There is so much to learn, so much to know. You all should know French and German well and read them with facility and be able to talk in them too. Every one of us needs culture of the heart—not the one you may be thinking of, which ought to be kept in cold storage for some years yet, but the heart for great literature and great achievements with which even the average student should have a close acquaintance. In conclusion, I should like to quote you the words of Robert Louis Stevenson:—

> Contend my soul for moments and for hours.
> Each is with service pregnant; each reclaimed,
> Is like a kingdom conquered where to reign.

